# Thyroid cancer 1 (C8orf4) shows high expression, no mutation and reduced methylation level in lung cancers, and its expression correlates with β-catenin and DNMT1 expression and poor prognosis

**DOI:** 10.18632/oncotarget.16877

**Published:** 2017-04-06

**Authors:** Yi-Wen Zheng, Li Zhang, Yuan Wang, Song-Yan Chen, Lei Lei, Na Tang, Da-Lei Yang, Lin-Lin Bai, Xiu-Peng Zhang, Gui-Yang Jiang, Lian-He Yang, Hong-Tao Xu, Qing-Chang Li, Xue-Shan Qiu, En-Hua Wang

**Affiliations:** ^1^ Department of Pathology, The First Affiliated Hospital and College of Basic Medical Sciences of China Medical University, Shenyang 110001, China

**Keywords:** C8orf4, mutation, methylation, β-catenin, DNMT1

## Abstract

Thyroid cancer 1 (TC1, C8orf4) plays important roles in tumors. The aim of this study was to examine the protein expression levels, methylation status, and mutational status of *TC1* (*C8orf4*) in lung cancers, and investigate the correlation between TC1, other members of the Wnt signaling pathway, and lung cancer. TC1 expression levels were assessed via immunohistochemical staining in 179 cases of lung cancer. β-catenin, TCF4, Axin, Disabled-2, Chibby, and DNA methyltransferase-1 (DNMT1) expressions were also examined. Bisulfite sequencing PCR analysis was used to examine the methylation status of the *C8orf4* locus, while PCR analysis and direct sequencing were used to determine its mutational status. We found high TC1 expression correlated with poor differentiation, advanced TNM stage, lymphatic metastasis, and poor prognosis in lung cancer patients. TC1 expression also correlated with β-catenin and DNMT1 expressions. No mutations in *C8orf4* were detected. However, methylation levels of *C8orf4* in lung cancers were lower than in corresponding normal lung tissues. In conclusion, high TC1 expression is implicated in lung cancer progression and correlates with poor prognosis in lung cancer. Reduced methylation levels might be responsible for the elevated TC1 expression levels. TC1, β-catenin, and DNMT1 can synergistically activate Wnt/β-catenin signaling in lung cancers.

## INTRODUCTION

Thyroid cancer 1 (TC1, C8ofr4) was originally identified in papillary thyroid cancer and its surrounding normal thyroid tissue less than two decades ago [1, 2]. It has multiple functions and is believed to play important roles in cell cycle control as well as transcriptional and translational regulation [1–5]. Several studies reported that TC1 functions as a regulator of the Wnt/β-catenin signaling pathway through its interactions with Chibby [4]. Chibby is a conserved nuclear protein that binds β-catenin and antagonizes β-catenin-mediated transcription [6–8]. TC-1 interacts with Chibby via its transient helical structure, and thereby enhances the Wnt/β-catenin signaling pathway by releasing β-catenin from Chibby. Free nuclear β-catenin then forms a complex with transcription factors of the T-cell factor/lymphoid enhancer factor (TCF/LEF) family, leading to the activation of Wnt target genes [9]. Thus, TC1 is considered a positive regulator of the Wnt signaling pathway [4, 10, 11]. Besides regulating the Wnt/β-catenin signaling pathway, TC1 is also a NF-κB-target gene and can be up-regulated by transforming growth factor β pathway and the IL-1β/TNF-α and fibroblast growth factor receptor 2 pathways [3, 5, 12, 13]. Heat shock and various cellular stresses can induce the expression of TC1, and TC1 serves as a novel heat shock response regulator [14]. TC1 is involved in the mitogen-activated ERK1/2 signaling pathway and promotes the G1- to S-phase transition of the cell cycle [15]. Recently, TC1 was also reported as a novel hematopoietic regulator in mice [16].

Although originally identified in papillary thyroid cancer, TC1 is ubiquitously expressed in human tissues [2]. Studies have shown that TC1 expression levels positively correlates with development of many malignancies including thyroid cancer [1, 17], gastric cancer [10], breast cancer [13], ovarian carcinomas [18], oral tongue squamous cell carcinomas [19], and hematological malignancies [20, 21]. Expression levels of TC1 correlate with poor clinical outcome and decreased survival in patients with gastric cancer and hematological malignancies [10, 20]. However, reduced expression of TC1 has been reported in colon cancer tissue relative to normal mucosa [3]. A recent report by Zhu et al. demonstrated that TC1 negatively regulated self-renewal of liver cancer stem cells via suppression of Notch2 signaling, thereby implicating a negative role for TC1 in tumor development [22]. Thus, understanding the precise functions and regulatory mechanisms of TC1 in cancers warrants further investigation.

Very little information exists regarding the role of TC1 in lung cancers [23, 24]. The regulatory mechanisms of TC1 expression, and its implications on clinicopathological factors and on outcomes of lung cancer patients remain unclear. In this study, we examined the protein expression levels, gene methylation status, and mutational status of *TC1* in lung cancer samples and explored the mechanisms underlying TC1 overexpression. We also investigated the correlation between expression levels of TC1 and other members of the Wnt/β-catenin signaling pathway, and analyzed the implications of TCI levels on clinicopathological factors and prognosis of lung cancer patients.

## RESULTS

### High TC1 expression correlates with poor differentiation, advanced TNM stage, lymphatic metastasis and poor prognosis in lung cancers

**Figure 1:**
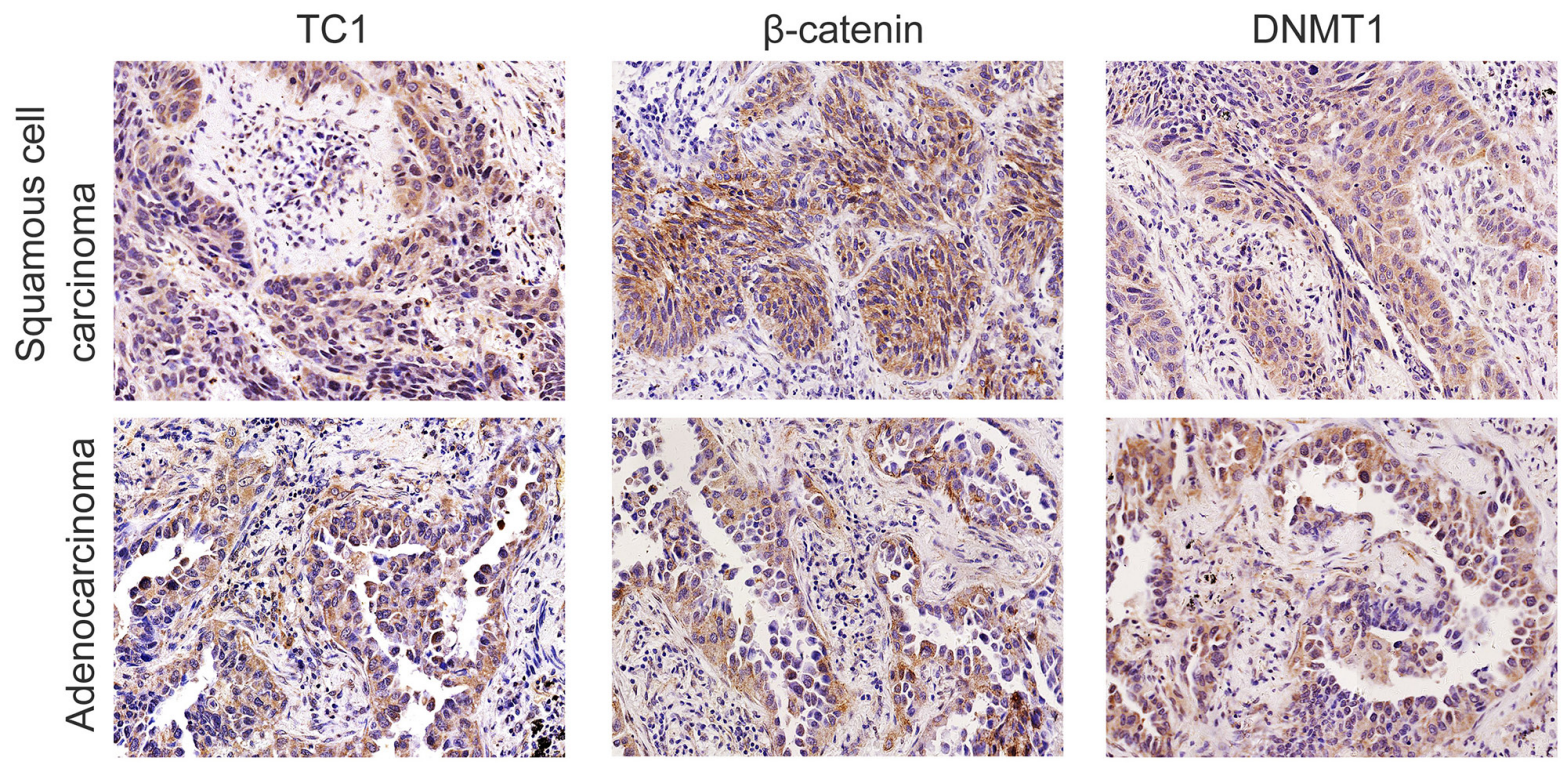
Expressions of TC1, β-catenin, and DNMT1 in representative lung cancer cases In a lung squamous cell carcinoma case, TC1 was highly expressed in the cytoplasm. Some cancer cells also showed nuclear staining of TC1. Expression of β-catenin and DNMT1 were both positive in the same case. In another adenocarcinoma case, TC1 was highly expressed in the cytoplasm. Some cancer cells also showed nuclear staining of TC1. Expression of β-catenin and DNMT1 were both positive in the same case (Original magnification, 200×; streptavidin-peroxidase immunohistochemistry method).

High TC1 expression was detected in 116 of 179 lung cancer cases. TC1 expression was primarily cytoplasmic with positive nuclear expression detected in 17 cases (Figure 1). As shown in Table 1, the high TC1 expression correlated with poor differentiation (*P* = 0.008), advanced TNM stage (*P* = 0.003) and lymphatic metastasis (*P* = 0.003) of lung cancers. TC1 expression did not correlate with the patients’ sex (*P* = 0.257), age (*P* = 0.776) or histological type of lung cancers (*P* = 0.210).

**Table 1:** The correlation between the expression of TC1 and clinicopathologycal factors of lung cancers

	n	TC1 cytoplasmic expression			TC1 nuclear expression		
		Low	High	*P* value	Negative	Positive	*P* value
Sex				0.257			0.707
Male	92	36	56		84	8	
Female	87	27	60		78	9	
Age				0.776			0.723
< 60	102	35	67		93	9	
≥ 60	77	28	49		69	8	
Histological type				0.210			0.600
Squamous cell carcinoma	63	26	37		58	5	
Adenocarcinoma	116	37	79		104	12	
Differentiation				0.008			0.745
Well	52	27	25		46	6	
Moderate	100	30	70		92	8	
Poor	27	6	21		24	3	
TNM stage				0.003			0.082
I	87	41	46		83	4	
II	72	19	53		62	10	
III	20	3	17		17	3	
Lymphatic metastasis				0.003			0.183
No	101	45	56		94	7	
Yes	78	18	60		68	10	

**Figure 2:**
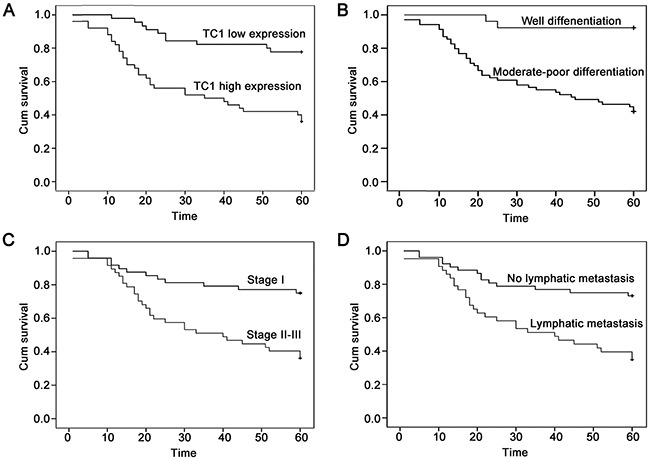
The Kaplan-Meier curves of lung cancer patients **(A)** The Kaplan-Meier curve of lung cancer patients with high or low TC1 expression. **(B)** The Kaplan-Meier curve of lung cancer patients with well or moderate-poor differentiation. **(C)** The Kaplan-Meier curve of lung cancer patients with TNM stage I or stage II-III. **(D)** The Kaplan-Meier curve of lung cancer patients with or without lymphatic metastasis.

A follow-up analysis was performed with 95 lung cancer patients. Kaplan-Meier curve and log rank analysis demonstrated that the average survival time of patients with high TC1 expression (36.220 ± 3.192) was shorter than that of patients with low TC1 expression (52.800 ± 2.210) (*P* < 0.001) (Figure 2). Poor differentiation (*P* < 0.001), advanced TNM stage (*P* < 0.001), and lymphatic metastasis (*P* < 0.001) also correlated with a lower survival rate (Figure 2). Further, multivariate Cox regression analysis (method: Forward Stepwise) revealed that high expression levels of TC1 (*P* = 0.001; hazard ratio: 3.376; 95% CI: 1.632-6.985) and lymphatic metastasis (*P* = 0.009; hazard ratio: 2.416; 95% CI: 1.252-4.661) were independent prognostic factors for lung cancer patients.

### Expression of TC1 correlates with the expressions of β-catenin, DNMT1, and Chibby

As shown in Table 2, along with the examination of TC1, expression levels of DNMT1, β-catenin, TCF4, Axin, Dab2, and Chibby were also examined in 84 lung cancer specimens. High cytoplasmic expression of TC1 positively correlated with expression levels of DNMT1 (*P* < 0.001; correlation coefficient = 0.502) and β-catenin (*P* = 0.003; correlation coefficient = 0.324) (Figure 1). Furthermore, β-catenin expression levels also correlated with the expression of DNMT1 (*P* = 0.020; correlation coefficient = 0.254). Thus, the expressions levels of TC1, β-catenin and DNMT1 correlated with each other. In addition, high cytoplasmic expression of TC1 negatively correlated with nuclear expression of Chibby (*P* = 0.001; correlation coefficient = −0.353). Nuclear expression of TC1 correlated with nuclear expressions of Axin (*P* = 0.004; correlation coefficient = 0.313) and Dab2 (*P* < 0.001; correlation coefficient = 0.399). Detailed correlative data of the examined proteins are listed in the Supplementary Table 1.

**Table 2:** The correlations between the expression of TC1 and DNMT1 or members of Wnt signaling pathway in lung cancers

	TC1 cytoplasmic expression					TC1 nuclear expression			
	n	Low	High	Correlationcoefficient	*P* value	Negative	Positive	Correlationcoefficient	*P* value
DNMT1				0.502	<0.001			−0.69	0.534
Low	31	15	16			26	5		
High	53	3	50			47	6		
β-catenin				0.324	0.003			0.113	0.305
Low	35	13	22			32	3		
High	49	5	44			41	8		
TCF4				0.063	0.572			−0.153	0.164
Low	37	9	28			30	7		
High	47	9	38			43	4		
Cytoplasmic Axin				0.017	0.879			−0.104	0.345
Low		11	39			42	8		
High		7	27			31	3		
Nuclear Axin				0.061	0.584			0.313	0.004
Negative	66	15	51			61	5		
Positive	18	3	15			12	6		
Cytoplasmic Disabled-2				0.152	0.166			−0.045	0.682
Low	26	8	18			22	4		
High	58	10	48			51	7		
Nuclear Disabled-2				−0.123	0.265			0.399	<0.001
Negative	56	10	46			54	2		
Positive	28	8	20			19	9		
Cytoplasmic Chibby				0.052	0.635			−0.138	0.210
Low	7	2	5			5	2		
High	77	16	61			68	9		
Nuclear Chibby				−0.353	0.001			−0.133	0.226
Negative	55	6	49			46	9		
Positive	29	12	17			27	2		

Underline: statistical significant.

### C8orf4 is not mutated in lung tumors or normal lung tissues

**Figure 3:**
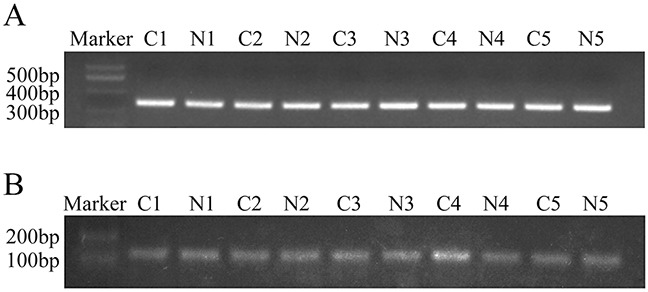
PCR analysis of the *C8orf4* gene locus and the CpG island of *C8orf4* after bisulfite conversion **(A)** The *C8orf4* gene was amplified and the product length was 315 bp. **(B)** After bisulfite conversion, the CpG island of *C8orf4* was amplified and the product length was 144 bp. C: lung cancer tissues. N: corresponding normal lung tissues.

**Figure 4:**
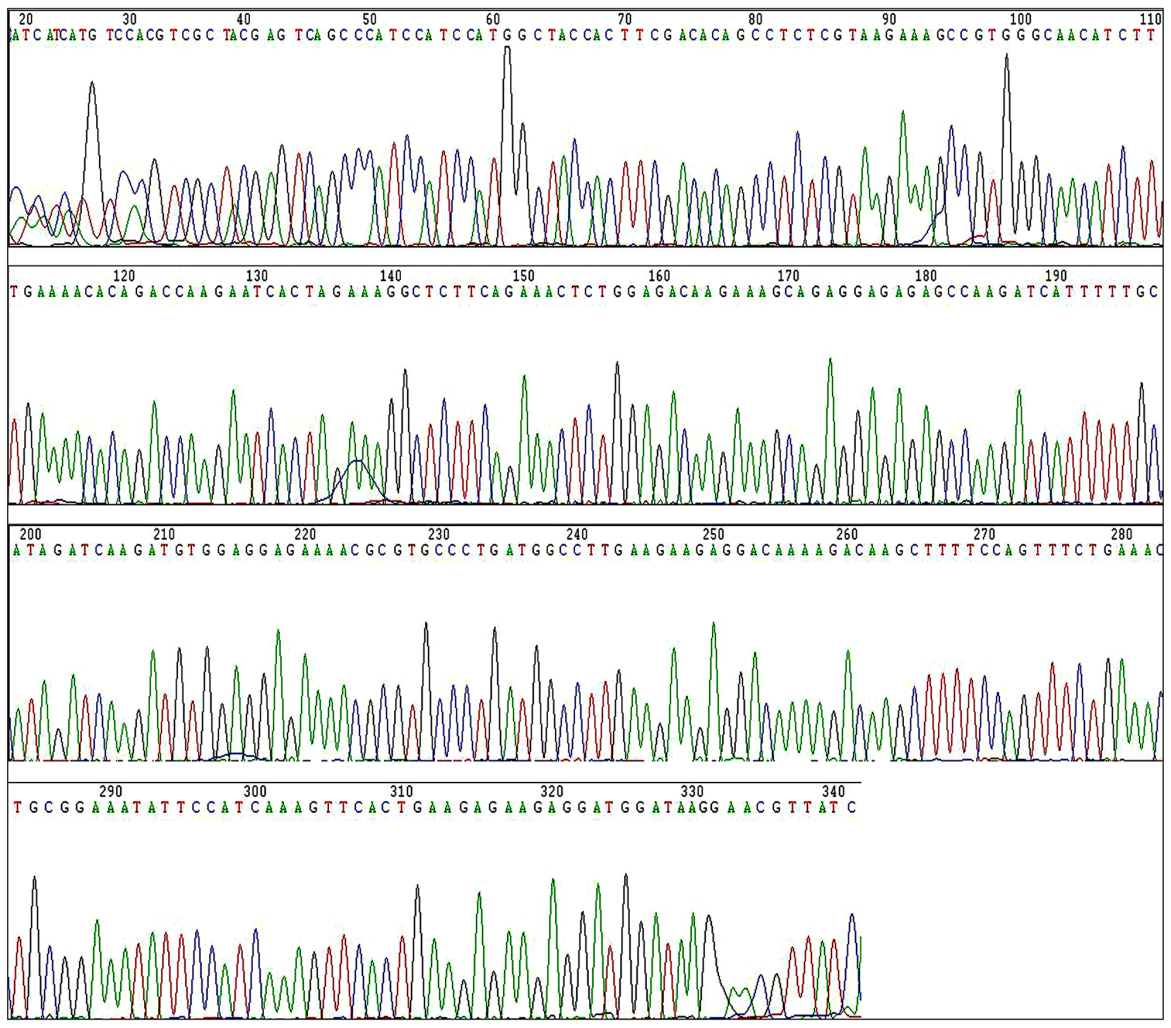
The result of sequencing of the *C8orf4* gene in a representative lung cancer case Sequencing of the *C8orf4* gene did not find any mutation in lung cancer tissues.

Sequence analysis of the *C8orf4* gene locus was performed on 70 lung cancer specimens as well as 30 normal lung tissues. Following PCR amplification and direct sequencing, no mutations in the *C8orf4* gene were detected in either lung cancer tissues or normal lung tissues (Figures 3A and 4).

### Methylation levels of C8orf4 in lung cancer specimens are lower than that in corresponding normal lung tissues

**Figure 5:**
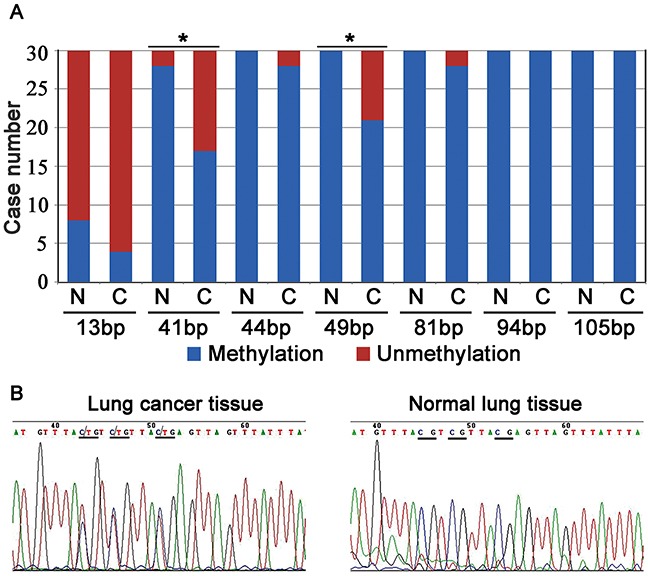
The results of Bisulfite sequencing PCR (BSP) analysis **(A)** The number of cases with methylation or unmethylation at the CpG sites in lung cancer tissues and their corresponding normal lung tissues. N: normal lung tissue; C: lung cancer tissue; *: *P* < 0.05. **(B)** In a representative lung cancer case, the CpG sites (underlined) at the 41 bp, 44 bp, and 49 bp were semi-methylated, whereas their corresponding sites in normal lung tissue were completely methylated (underlined).

We analyzed the gene sequence of *C8orf4* from −2000 bp to 1000 bp using the online CpG island predict program from The Li Lab (http://www.urogene.org/methprimer/index.html). The CpG island of the *C8orf4* gene was located closer to the 5′ end of the exon, near the start codon. After bisulfite sequencing PCR (BSP), the methylation status of each CpG site was examined in 30 pairs of lung cancer tissues and their corresponding normal lung tissues (Figure 3B). As indicated in Table 3 and Figure 5, complete methylation was observed at the CpG sites at 41 bp and 49 bp from the start codon, in 17 and 21 cases of lung cancer, respectively, which was less than that in corresponding normal lung tissues (28 cases for the 41 bp site [*P* = 0.001] and 30 for the 49 bp site [*P* = 0.003]). Complete methylation of CpG sites at 94 bp and 105 bp was observed in all lung cancer samples and their corresponding normal lung tissues. Although the levels of complete methylation at CpG sites of 13 bp (*P* = 0.102), 44 bp (*P* = 0.157), and 81 bp (*P* = 0.157) were lower in lung cancer tissues than that in their corresponding normal lung tissues, these differences were not statistically significant. Furthermore, the methylation status of the 49 bp CpG site correlated with those of the 41 bp (*P* < 0.001; correlation coefficient = 0.749) and 44 bp (*P* = 0.408; correlation coefficient = 0.025) CpG sites, and the methylation status of the 44 bp CpG site correlated with the 81 bp CpG site (*P* = 0.010; correlation coefficient = 0.464). Correlation between the methylation status and clinicopathological factors in lung cancer specimens was also investigated, but no statistically significant results were observed (data not shown).

**Table 3:** The methylation status of TC1 in lung cancers and corresponding normal lung tissues

		The methylation status of TC1		
The CpG site	Methylation	Unmethylation	Wilcoxon signedranks test	*P* value
13bp			1.633	0.102
Lung cancer	4	26		
NL	8	22		
41bp			3.317	0.001
Lung cancer	17	13		
NL	28	2		
44bp			1.414	0.157
Lung cancer	28	2		
NL	30	0		
49bp			3.000	0.003
Lung cancer	21	9		
NL	30	0		
81bp				
Lung cancer	28	2	1.414	0.157
NL	30	0		
94bp			0.000	1.000
Lung cancer	30	0		
NL	30	0		
105bp			0.000	1.000
Lung cancer	30	0		
NL	30	0		

NL: corresponding normal lung tissue.

Underline: statistical significant.

## DISCUSSION

Several studies reported high expression levels of TC1 across a wide range of tumors, and a positive association with malignancy [10, 13, 17–19, 23, 24]. However, reduced expression of TC1 was observed in colon cancer tumors relative to normal mucosa [3]. TC1 likely exhibits pro- or anti-tumorigenic roles in a tumor type-specific manner. Consistent with previous reports, our study demonstrated high expression levels of TC1 correlates with the tumor development and decreased survival, and is a predictor of poor prognosis in lung cancer patients.

Because the mechanism underlying abnormal TC1 expression in tumors had remained unclear, we hypothesized that gene methylation or mutations in *TC1* contribute to the high expression level in lung cancer. To our knowledge, this is the first study examining the methylation status as well as mutational status of the *C8orf4* gene in tumors. No mutation in the *C8orf4* locus was detected in either lung tumor specimens or the corresponding normal lung tissues, suggesting a mechanism other than mutation in TC1 contributes to high expression levels in lung cancer. BSP analysis revealed that the degree of methylation at the 41 bp and 49 bp CpG sites in lung cancer tissues was lower than that in corresponding normal lung tissues. These results suggest that reduced methylation levels contribute to high expression of TC1 in lung cancers, and that the 41 bp and 49 bp CpG sites serve as key methylation sites that likely modulate the expression levels of TC1.

It is believed that TC1 promotes tumor development primarily by enhancing the activity of the Wnt/β-catenin signaling pathway [10, 13, 19, 23]. However, the relationship between TC1 and other key members of the Wnt/β-catenin signaling pathway is unclear. We thus examined the correlation between TC1 and other Wnt pathway members including β-catenin, Chibby, TCF4, Axin, and Dab2 in lung cancers. The expression of TC1 positively correlates with the expression of β-catenin, and negatively correlates with the nuclear expression of Chibby. These data provided further evidence that TC1 inhibits the function of nuclear Chibby while enhancing the activity of β-catenin. We also observed that nuclear expression of TC1 correlated with nuclear expression of Axin and Dab2, which are both important inhibitors of the Wnt/β-catenin signaling pathway [25–27]. Since the number of cases containing TC1 nuclear expression was very small, these results need further confirmation. Recent report revealed that DNMT1 expression correlates with β-catenin expression, colocalizes with β-catenin, and regulates the activity of the Wnt/β-catenin signaling pathway [8, 28]. The present study observed that the expression of TC1, β-catenin, and DNMT1 were correlated with each other, indicating that these three proteins might promote the activity of the Wnt signaling pathway in a synergistic manner. Recent studies revealed that TC1 plays a role in the regulation of stem cells, such as hematopoietic stem cells [16], adipocyte-derived stem cells [29], and liver cancer stem cells [22] via the Wnt/β-catenin signaling pathway or Notch signaling pathway. Therefore, TC1 may participate in tumorigenesis and the transformation of cancer stem cells, which require further studies.

In summary, high TC1 expression is implicated in lung cancer progression and correlates with poor prognosis in lung cancer patients. Our data indicated that reduced methylation levels, rather than gene mutation, might be the underlying cause for high TC1 expression in lung cancer. Finally, our results further suggested that TC1, β-catenin and DNMT1 might promote the Wnt signaling activity in a synergistic manner.

## MATERIALS AND METHODS

### Patients and tissue specimens

In this study, 179 lung squamous cell carcinoma (SCC) and adenocarcinoma samples were obtained randomly from patients who underwent surgery in the First Affiliated Hospital of China Medical University between 2007 and 2013. Among the 179 patients, 95 had complete follow-up records. The patient pool comprised of 92 men and 87 women, with a mean age of 60 years (range, 38–84 years). Histological diagnosis and grade of differentiation were determined by examination of hematoxylin-eosin stained sections in accordance with the classification system of the World Health Organization. Tumors were diagnosed as SCCs (*n* = 63) or adenocarcinomas (*n* = 116), and classified as well-differentiated (*n* = 52), moderately differentiated (*n* = 100) or poorly differentiated (*n* = 27) tumors. Tumor stage was classified as stages I–III (*n* = 87, 72 and 20, respectively) according to the TNM classification system of the International Union Against Cancer. Lymphatic metastasis was observed in 78 cases. Seventy fresh lung cancer samples and 30 of their corresponding normal lung tissues were also obtained from patients who underwent surgery at the First Affiliated Hospital of China Medical University in 2013 and stored at −70°C immediately after resection. These samples were used for DNA extraction. The study was conducted under the regulations of the Institutional Review Board of China Medical University.

### Immunohistochemistry

Specimens were fixed in 10% neutral-buffered formalin for 24 hours, embedded in paraffin blocks and cut into 4-μm sections. These sections were deparaffinized, rehydrated, and then incubated with rabbit anti-TC1 antibody (1:200; Santa Cruz, CA), mouse anti-DNA methyltransferase-1 (DNMT1) antibody (1:200; Santa Cruz, CA), mouse anti-β-catenin antibody (1:200; BD Transduction Laboratories, KY), rabbit anti-TCF4 antibody (1:200; Santa Cruz, CA), rabbit anti-Axin antibody (1:50; Santa Cruz, CA), rabbit anti-Disabled-2 (Dab2) antibody (1:200; Santa Cruz, CA) or rabbit anti-Chibby antibody (1:200; Santa Cruz, CA) at 4°C overnight. Detection of antibodies was performed using the streptavidin-peroxidase method. Some slides were stained in the absence of primary antibodies and served as negative controls.

### Evaluation of immunostaining

Two investigators who were blind to the clinical data evaluated the immunostained sections. Five views per slide were examined, and 100 tumor cells were observed per view at 400× magnification. The positive rate of each case was obtained by calculating the percentage of positively stained cells in each slide. Percentage score of each case was assigned as follows: (1) 1–25%, (2) 26–50%, (3) 51–75%, and (4) 76–100%. The intensity of immunostaining was scored as 0, 1, 2, or 3 if negative, weak, moderate, or marked, respectively. Scores from each tumor sample were multiplied to give a final score ranging 0-12, and the tumors were categorized based on scores ≤6 and ≥8 as having low or high expression, respectively. When ≥10% of tumor cells per specimen were stained in the nuclear regions, the sample was scored as having positive nuclear expression.

### DNA extraction, PCR, and direct sequencing

Genomic DNA was isolated from tissue samples with a tissue/cell DNA extraction reagent kit (Bioteke, Beijing, China) according to the manufacturer's protocol. The primers for *C8orf4* gene were as follows: forward, 5′-CGATGAAAGCAAAGCGAAGCC -3′, and reverse, 5′- GATAACGTCCTTATCCATCCTC -3′. PCR conditions included denaturation at 95°C for 5 min, amplification for 30 cycles at 95°C for 20 s, annealing at 55°C for 20 s, and extension at 72°C for 40 s, with a final step at 4°C for 5 min. The PCR product of *C8orf4* was 351 bp. PCR products were analyzed via electrophoresis on 1% agarose gels containing ethidium bromide, and observed using a Bio-Imaging system (UVP, Upland, CA). Purified PCR products were sequenced by Wanlei Life Sciences Co., Ltd., Shenyang, China.

### Bisulfite sequencing PCR (BSP) analysis

Following DNA extraction, bisulfite conversion of DNA was performed using the EZ DNA Methylation kit (Zymo Research, Beijing, China) according to the manufacturer's instructions. The primers for BSP were as follows: forward, 5′-GGAGTTGAATTTCGGAAGAT-3′; reverse, 5′-ATTACCCACGACTTTCTTAC-3′ (product length: 144 bp). The bisulfite-treated DNA was amplified for 30 cycles: 95°C for 5 min, followed by cycling at 95°C for 10 s, 52°C for 20 s, and 72°C for 30 s, with a final step at 4°C for 5 min. PCR products were analyzed using electrophoresis on 1.5% agarose gels containing ethidium bromide, and observed using a Bio-Imaging system (UVP, Upland, CA). Purified PCR products were sequenced by Wanlei Life Sciences Co., Ltd., Shenyang, China.

### Statistical analysis

Pearson's chi-square and likelihood ratio tests were used to examine correlations between TC1 expression and clinicopathological factors. Spearman's correlation test was used to assay correlations between expression levels of these proteins. Non-parametric Wilcoxon signed ranks test was used to compare the difference between TC1 methylation status of lung cancers and the corresponding normal lung tissues. Kaplan–Meier method (log-rank test) and Multivariate Cox regression were used in follow-up analysis. Statistical significance was established at *P*<0.05.

## SUPPLEMENTARY TABLE





## Abbreviations

TC1: thyroid cancer 1
DNMT1: DNA methyl-transferase-1
TCF/LEF: T-cell factor/lymphoid enhancer factor
Dab2: Disabled-2
SCC: squamous cell carcinoma
BSP: bisulfite sequencing PCR.
