# Supplementary material for: Thyroid cancer 1 (C8orf4) shows high expression, no mutation and reduced methylation level in lung cancers, and its expression correlates with β-catenin and DNMT1 expression and poor prognosis
Source: Oncotarget. 2017 Apr 6;8(38):62880–90. doi: 10.18632/oncotarget.16877 (PMC5609888; doi:10.18632/oncotarget.16877)
Supplement: Supplementary file 1 [file oncotarget-08-62880-s001.pdf]

Thyroid cancer 1 (C8orf4) shows high expression, no mutation and reduced methylation level in lung cancers, and its expression correlates with β-catenin and DNMT1 expression and poor prognosis

SUPPLEMENTARY TABLE

Supplementary Table 1: The correlations among the expressions of TC1, DNMT1, β-catenin, TCF4, Axin, Dab2, and Chibby.

See Supplementary File 1
