# Supplementary material for: Thyroid cancer 1 (C8orf4) shows high expression, no mutation and reduced methylation level in lung cancers, and its expression correlates with β-catenin and DNMT1 expression and poor prognosis
Source: Oncotarget. 2017 Apr 6;8(38):62880–90. doi: 10.18632/oncotarget.16877 (PMC5609888; doi:10.18632/oncotarget.16877)
Supplement: Supplementary file 2 [file oncotarget-08-62880-s002.doc]

**Supplementary Table 1: The correlations among the expressions of TC1, DNMT1, β-catenin, TCF4, Axin, Dab2, and Chibby.**

|  | | | Cytoplasmic TC1 | nuclear TC1 | DNMT1 | beta-catenin | nuclear beta-catenin | Cytoplasmic TCF4 | nuclear TCF4 | Cytoplasmic Axin | nuclear Axin | Cytoplasmic Dab2 | nuclear Dab2 | Cytoplasmic Cby | nuclear Cby |
| --- | --- | --- | --- | --- | --- | --- | --- | --- | --- | --- | --- | --- | --- | --- | --- |
| Spearman's rho | CytoplasmicTC1 | Correlation Coefficient | 1.000 | 0.159 | 0.502 | 0.324 | 0.181 | 0.063 | -0.116 | 0.017 | 0.061 | 0.152 | -0.123 | 0.052 | -0.353 |
|  |  | Sig. (2-tailed) | . | 0.034 | 0.000 | 0.003 | 0.100 | 0.572 | 0.293 | 0.879 | 0.584 | 0.166 | 0.265 | 0.635 | 0.001 |
|  |  | N | 179 | 179 | 84 | 84 | 84 | 84 | 84 | 84 | 84 | 84 | 84 | 84 | 84 |
|  | nuclear TC1 | Correlation Coefficient | 0.159 | 1.000 | -0.069 | 0.113 | 0.094 | -0.153 | -0.106 | -0.104 | 0.313 | -0.045 | 0.399 | -0.138 | -0.133 |
|  |  | Sig. (2-tailed) | 0.034 | . | 0.534 | 0.305 | 0.396 | 0.164 | 0.338 | 0.345 | 0.004 | 0.682 | 0.000 | 0.210 | 0.226 |
|  |  | N | 179 | 179 | 84 | 84 | 84 | 84 | 84 | 84 | 84 | 84 | 84 | 84 | 84 |
|  | DNMT1 | Correlation Coefficient | 0.502 | -0.069 | 1.000 | 0.254 | 0.105 | -0.033 | -0.074 | 0.078 | -0.082 | 0.128 | -0.140 | 0.037 | -0.327 |
|  |  | Sig. (2-tailed) | 0.000 | 0.534 | . | 0.020 | 0.340 | 0.769 | 0.503 | 0.482 | 0.461 | 0.245 | 0.205 | 0.737 | 0.002 |
|  |  | N | 84 | 84 | 84 | 84 | 84 | 84 | 84 | 84 | 84 | 84 | 84 | 84 | 84 |
|  | beta-catenin | Correlation Coefficient | 0.324 | 0.113 | 0.254 | 1.000 | 0.137 | 0.126 | 0.024 | 0.057 | -0.147 | 0.113 | -0.171 | 0.182 | 0.004 |
|  |  | Sig. (2-tailed) | 0.003 | 0.305 | 0.020 | . | 0.215 | 0.255 | 0.827 | 0.604 | 0.182 | 0.305 | 0.120 | 0.098 | 0.970 |
|  |  | N | 84 | 84 | 84 | 84 | 84 | 84 | 84 | 84 | 84 | 84 | 84 | 84 | 84 |
|  | nuclear beta-catenin | Correlation Coefficient | 0.181 | 0.094 | 0.105 | 0.137 | 1.000 | 0.152 | -0.038 | -0.050 | -0.087 | -0.018 | 0.082 | 0.104 | -0.252 |
|  |  | Sig. (2-tailed) | 0.100 | 0.396 | 0.340 | 0.215 | . | 0.167 | 0.728 | 0.649 | 0.431 | 0.872 | 0.460 | 0.344 | 0.021 |
|  |  | N | 84 | 84 | 84 | 84 | 84 | 84 | 84 | 84 | 84 | 84 | 84 | 84 | 84 |
|  | Cytoplasmic TCF4 | Correlation Coefficient | 0.063 | -0.153 | -0.033 | 0.126 | 0.152 | 1.000 | 0.504 | 0.243 | -0.063 | 0.184 | -0.034 | 0.253 | -0.011 |
|  |  | Sig. (2-tailed) | 0.572 | 0.164 | 0.769 | 0.255 | 0.167 | . | 0.000 | 0.026 | 0.572 | 0.094 | 0.759 | 0.020 | 0.918 |
|  |  | N | 84 | 84 | 84 | 84 | 84 | 84 | 84 | 84 | 84 | 84 | 84 | 84 | 84 |
|  | nuclear TCF4 | Correlation Coefficient | -0.116 | -0.106 | -0.074 | 0.024 | -0.038 | 0.504 | 1.000 | -0.049 | 0.058 | 0.103 | 0.152 | 0.215 | 0.075 |
|  |  | Sig. (2-tailed) | 0.293 | 0.338 | 0.503 | 0.827 | 0.728 | 0.000 | . | 0.661 | 0.600 | 0.351 | 0.169 | 0.049 | 0.497 |
|  |  | N | 84 | 84 | 84 | 84 | 84 | 84 | 84 | 84 | 84 | 84 | 84 | 84 | 84 |
|  | Cytoplasmic Axin | Correlation Coefficient | 0.017 | -0.104 | 0.078 | 0.057 | -0.050 | 0.243 | -0.049 | 1.000 | -0.135 | 0.395 | -0.171 | 0.073 | 0.013 |
|  |  | Sig. (2-tailed) | 0.879 | 0.345 | 0.482 | 0.604 | 0.649 | 0.026 | 0.661 | . | 0.220 | 0.000 | 0.119 | 0.509 | 0.904 |
|  |  | N | 84 | 84 | 84 | 84 | 84 | 84 | 84 | 84 | 84 | 84 | 84 | 84 | 84 |
|  | nuclear Axin | Correlation Coefficient | 0.061 | 0.313 | -0.082 | -0.147 | -0.087 | -0.063 | 0.058 | -0.135 | 1.000 | 0.161 | 0.431 | -0.052 | -0.074 |
|  |  | Sig. (2-tailed) | 0.584 | 0.004 | 0.461 | 0.182 | 0.431 | 0.572 | 0.600 | 0.220 | . | 0.143 | 0.000 | 0.635 | 0.503 |
|  |  | N | 84 | 84 | 84 | 84 | 84 | 84 | 84 | 84 | 84 | 84 | 84 | 84 | 84 |
|  | Cytoplasmic Dab2 | Correlation Coefficient | 0.152 | -0.045 | 0.128 | 0.113 | -0.018 | 0.184 | 0.103 | 0.395 | 0.161 | 1.000 | 0.091 | 0.264 | -0.001 |
|  |  | Sig. (2-tailed) | 0.166 | 0.682 | 0.245 | 0.305 | 0.872 | 0.094 | 0.351 | 0.000 | 0.143 | . | 0.410 | 0.015 | 0.991 |
|  |  | N | 84 | 84 | 84 | 84 | 84 | 84 | 84 | 84 | 84 | 84 | 84 | 84 | 84 |
|  | nuclear Dab2 | Correlation Coefficient | -0.123 | 0.399 | -0.140 | -0.171 | 0.082 | -0.034 | 0.152 | -0.171 | 0.431 | 0.091 | 1.000 | 0.030 | -0.142 |
|  |  | Sig. (2-tailed) | 0.265 | 0.000 | 0.205 | 0.120 | 0.460 | 0.759 | 0.169 | 0.119 | 0.000 | 0.410 | . | 0.783 | 0.199 |
|  |  | N | 84 | 84 | 84 | 84 | 84 | 84 | 84 | 84 | 84 | 84 | 84 | 84 | 84 |
|  | Cytoplasmic Cby | Correlation Coefficient | 0.052 | -0.138 | 0.037 | 0.182 | 0.104 | 0.253 | 0.215 | 0.073 | -0.052 | 0.264 | 0.030 | 1.000 | 0.219 |
|  |  | Sig. (2-tailed) | 0.635 | 0.210 | 0.737 | 0.098 | 0.344 | 0.020 | 0.049 | 0.509 | 0.635 | 0.015 | 0.783 | . | 0.045 |
|  |  | N | 84 | 84 | 84 | 84 | 84 | 84 | 84 | 84 | 84 | 84 | 84 | 84 | 84 |
|  | nuclear Cby | Correlation Coefficient | -0.353 | -0.133 | -0.327 | 0.004 | -0.252 | -0.011 | 0.075 | 0.013 | -0.074 | -0.001 | -0.142 | 0.219 | 1.000 |
|  |  | Sig. (2-tailed) | 0.001 | 0.226 | 0.002 | 0.970 | 0.021 | 0.918 | 0.497 | 0.904 | 0.503 | 0.991 | 0.199 | 0.045 | . |
|  |  | N | 84 | 84 | 84 | 84 | 84 | 84 | 84 | 84 | 84 | 84 | 84 | 84 | 84 |

*. Correlation is significant at the 0.05 level (2-tailed).

**. Correlation is significant at the 0.01 level (2-tailed).
